# PLS-Based and Regularization-Based Methods for the Selection of Relevant Variables in Non-targeted Metabolomics Data

**DOI:** 10.3389/fmolb.2016.00035

**Published:** 2016-07-26

**Authors:** Renata Bujak, Emilia Daghir-Wojtkowiak, Roman Kaliszan, Michał J. Markuszewski

**Affiliations:** Department of Biopharmaceutics and Pharmacodynamics, Medical University of GdańskGdańsk, Poland

**Keywords:** statistical analysis, non-targeted metabolomics, mass spectrometry, orthogonal projections to latent structures-discriminant analysis, least absolute shrinkage and selection operator

## Abstract

Non-targeted metabolomics constitutes a part of the systems biology and aims at determining numerous metabolites in complex biological samples. Datasets obtained in the non-targeted metabolomics studies are high-dimensional due to sensitivity of mass spectrometry-based detection methods as well as complexity of biological matrices. Therefore, a proper selection of variables which contribute into group classification is a crucial step, especially in metabolomics studies which are focused on searching for disease biomarker candidates. In the present study, three different statistical approaches were tested using two metabolomics datasets (*RH* and *PH study*). The orthogonal projections to latent structures-discriminant analysis (OPLS-DA) without and with multiple testing correction as well as the least absolute shrinkage and selection operator (LASSO) with bootstrapping, were tested and compared. For the *RH study*, OPLS-DA model built without multiple testing correction selected 46 and 218 variables based on the VIP criteria using Pareto and UV scaling, respectively. For the *PH study*, 217 and 320 variables were selected based on the VIP criteria using Pareto and UV scaling, respectively. In the *RH study*, OPLS-DA model built after correcting for multiple testing, selected 4 and 19 variables as in terms of Pareto and UV scaling, respectively. For the *PH study*, 14 and 18 variables were selected based on the VIP criteria in terms of Pareto and UV scaling, respectively. In the *RH* and *PH study*, the LASSO selected 14 and 4 variables with reproducibility between 99.3 and 100%, respectively. In the light of PLS-based models, the larger the search space the higher the probability of developing models that fit the training data well with simultaneous poor predictive performance on the validation set. The LASSO offers potential improvements over standard linear regression due to the presence of the constrain, which promotes sparse solutions. This paper is the first one to date utilizing the LASSO penalized logistic regression in untargeted metabolomics studies.

## Introduction

Apart from genomics or proteomics, metabolomics is a relatively new and dynamically developing field of systems biology. Metabolomics is focused on qualitative and quantitative analysis of low-molecular-weight endogenous compounds in different biological matrices (urine, blood, tissue extracts) (Nicholson et al., 1999; Fiehn, 2001). Metabolome, analogously to a well-defined genome or proteome, covers all metabolites present in cells, tissues being under continuous change in physiological and pathophysiological conditions.

There are two research approaches which have emerged in metabolomics: targeted and non-targeted strategy (Barderas et al., 2011). Targeted metabolomics, known as metabolic profiling, relies on the quantitative analysis of selected group of metabolites characterized by similar physicochemical properties (i.e., carbohydrates, amino acids, organic acids, nucleosides) or belonging to the same biochemical pathway (i.e., gluconeogenesis, citric acid cycle) (Dudley et al., 2010). Non-targeted metabolomics is based on the qualitative measurement and comparison of as many metabolites as possible. Most commonly, both approaches are used to determine a wide spectrum (or subset) of metabolites in biological samples from different groups of individuals (e.g., healthy vs. diseased, responsive vs. non-responsive) or between different disease stages (cancer stage or grade) (Patti et al., 2012).

The data analysis methodology is strictly dependent on metabolomics research strategy. In the targeted approach, the number of samples is usually larger than the number of variables determined. Therefore, a method of choice is to use parametric (*t*-test) or non-parametric (Mann Whitney U test statistics) methods to check whether the concentration/levels of a particular metabolite significantly differs between the investigated groups. However, both targeted and untargeted approach is related to hypothesis testing if the goal is to select significant variables based on *p*-values. Since we usually test more than one hypothesis (or in other words, we determine the concentration/level of more than one metabolite), multiple testing adjustment should always be considered to control false positive results (Hovde, 2011; Vinaixa et al., 2012).

In non-targeted metabolomics studies in contrast, the number of variables highly exceeds the number of metabolic features detected. Therefore, the method of choice in high-dimensional and multicolinear metabolomics data is the use of (i) unsupervised methods such as the principal component analysis (PCA) as well as (ii) supervised discriminant techniques, such as the partial least squares-discriminant analysis (PLS-DA) and the orthogonal projections to latent structures-discriminant analysis (OPLS-DA) (Xi et al., 2014; Alonso et al., 2015).

The use of the above-mentioned techniques has been widely reported in metabolomics. However, despite their usefulness when analyzing high-dimensional data, the quality and predictive performance of the models developed are often poor due to model overfitting (Hendriks et al., 2011). Another drawback of the PLS-based methods is that they do not provide any statistical significance of variables expressed by *p*-values. Instead, the variable importance (VIP) measure is used to analyze the loadings which reflect the influence of each variable on the response. The VIP values greater than one are considered important and affect classification between the groups.

To prevent overfitting of the model, the number of variables should be reduced. Feature selection methods have been widely described in the literature to reduce false discoveries, especially when dealing with high dimensional and multicollinear data space. Feature selection is considered the most crucial task prior to modeling because it reduces overfitting of the model enhancing its generalization, making the model less complex and easier to interpret simultaneously improving its performance (Goodarzi et al., 2012). Controlling false discovery rate (FDR) is a statistical approach which enables controlling the FDR of the features identified before developing PLS models (Goodacre et al., 2007; Bum Kim et al., 2008).

Apart from the PLS-based techniques for high-dimensional data space, an alternative approach which provides feature selection together with model development relates to regularization-based method, i.e., the Least Absolute Shrinkage and Selection Operator (LASSO). The LASSO has been reported to improve model performance in terms of multi-dimensional and multicollinear data analysis (Daghir-Wojtkowiak et al., 2015) and therefore, may be considered an alternative to commonly known PLS-based techniques.

The objective of this study was to test three different statistical approaches for the selection of variables which contributed the most into classification between the groups. Two datasets from untargeted LC/MS metabolomics studies were used. We developed models using (i) OPLS-DA without multiple testing correction, (ii) OPLS-DA with multiple testing correction, and (iii) LASSO regularization. Within the OPLS-DA analysis, we additionally compared the results in terms of the autoscaling (UV) and Pareto scaling. To the best authors' knowledge, this is the first study which demonstrates the concept of LASSO for the analysis of untargeted metabolomics data.

## Materials and methods

### Study design

In this study, three statistical approaches were tested using two datasets. The first dataset was denoted as the *RH study* and referred to a comparison between responsive (*n* = 81) and non-responsive (*n* = 69) hypertensive-treated patients. The compared groups were matched according to age (*p* = 0.79), body mass index (*p* = 0.28), and sex (*p* = 0.36).

The second dataset was denoted as the *PH study* and referred to a comparison between 20 patients suffered from pulmonary disease and 20 healthy individuals. The studied groups were matched according to age (*p* = 0.96), BMI (*p* = 0.87), and sex (*p* = 0.62).

In terms of the *RH study*, plasma samples' collection was performed according to the ethical agreement from an independent committee of bioethical research at the Medical University of Gdansk (NKEBN/285/2009). The *PH study* was carried out with the approval of the ethical committee of clinical investigations in Barcelona (CEIC, approval number CIF-G-08431173). Both studies were conducted with the understanding of the consent of each participant. All participants under study provided a written informed consent.

### Analytical measurements

Plasma metabolic fingerprinting in the *RH study* was performed with the Agilent 1200 Series LC system (Agilent Technologies, Waldbronn, Germany) coupled with the Agilent 6224 Series TOF LC/MS system (Agilent Technologies, Waldbronn, Germany). In the *PH study*, plasma metabolic fingerprinting was conducted with the Agilent 1200 Infinity series (Agilent Technologies, Waldbronn, Germany) coupled with the Agilent Technologies QTOF (6520) mass spectrometry detector. The chromatographic and mass spectrometer parameters of the optimized LC/MS methods were described in detail in the Supplementary Material section. Quality control samples (QCs) were prepared as a pool of equal volume of each plasma samples included in each study. The QCs were analyzed in order to monitor system's and method's stability during the whole sequence run. Detailed clinical information about studied groups in both non-targeted metabolomics studies were described in Tables S1, S2 in the Supplementary Material section.

### Data treatment, filtration, and normalization

The acquired chromatograms representing plasma metabolic fingerprints were extracted with the use of MFE algorithm provided by MassHunter Qualitative Analysis B.06.00 software (Agilent Technologies, Waldbronn, Germany). The parameters applied for data extraction were similar to the previously described (Ciborowski et al., 2014). The background noise threshold was set to 200 counts and the following adducts were included: +H, +Na, +K. Neutral water loss was also taken into account. After data extraction, each potential compound present in all plasma samples was described by the monoisotopic mass, retention time, and abundance.

Alignment of the chromatography data was performed with Mass Profiler Professional B.02.01 software (Agilent Technologies, Waldbronn, Germany) using 1% and 5 ppm for retention time and mass correction, respectively.

The aligned dataset was filtered based on the quality assurance (QA) criteria (Dunn et al., 2011) which included the presence of variables in at least 50% of QCs and the coefficient of variation (CV) value (< 20%) in QCs. A second filtering required the presence of the variable in 80% of the samples in at least one of the compared groups (i.e., in 80% of the samples in the responsive or non-responsive group, as well as in the *PH* patients or healthy individuals). These datasets were subsequently used for statistical analyses.

### Statistical methods

#### Orthogonal projections to latent structures (OPLS)-based methods

The partial least-squares (alternatively partial least squares projections to latent structures, PLS) is a latent variable regression method based on covariance between the predictors (*X*) and the response (*Y*) (Wold et al., 2001). A discriminant variant of PLS, particularly PLS-DA, refers to a classification method in which each observation is described by one out of two categories (Barker and Rayens, 2003). The PLS components are constrained to be orthogonal, the dimensionality-reducing transformation builds a matrix in which columns represent the first *P* eigenvectors of the matrix formed by the covariances between *X* and *Y* (Worley and Powers, 2013). Therefore, the PLS selects a subset of scores and loadings, namely the latent structures, which most effectively summarize *X* and *Y* describing correlation between them (Worley and Powers, 2013).

The implication of a class memberships in the PLS-DA provides better class separation in the scores space. Hence, variation which is not directly correlated with *Y* is still present in the scores (Worley and Powers, 2013). This makes interpretation of PLS-DA scores and loadings more complicated. The OPLS in turn simplifies this interpretation by incorporating the Orthogonal Signal Correction (OSC) filter into a PLS-based model and in consequence, the *Y* -predictive variation is effectively separated from the *Y* -uncorrelated variation in the *X* matrix (Sjoblom et al., 1998; Wold et al., 1998; Hoskuldsson, 2001).

The main difference between PLS-DA and OPLS-DA is that the latter one splits up the data variation into the variation related to *Y* and an orthogonal (noise) variation which is not related to *Y*. In turn it simplifies the interpretability of the obtained models providing an estimation of within- and between-group variability (Wiklund et al., 2008; Kim et al., 2009).

In this study, we developed two OPLS-DA models (i) without and (ii) with multiple testing correction using FDR (Benjamini-Hochberg 1995) procedure. Prior to model development, the normality of data distribution was assessed using the Shapiro-Wilk test followed by the application of parametric (*t*-test) or non-parametric (U Mann-Whitney test) tests. The homogeneity of variance between compared groups was checked with the use of the Levene's test and subsequently the standard *t*-test (in case of equal variances) or Welch's *t*-test (in case of unequal variances).

All statistical calculations regarding the OPLS-DA were performed using Matlab 2013b environment (Mathworks, Natick, MA, USA). The multivariate analyses and plottings were performed in SIMCA P+ 13.0.3 software (Umetrics, Umea, Sweden).

#### Scaling procedure

Scaling procedures are data pretreatment steps which divide each variable by the scaling factor, which has a different value for each variable (van den Berg et al., 2006). The aim of the data scaling is to adjust for fold differences between the measured variables (metabolite intensities) converting the data matrix relative to the scaling factor. Two subclasses of the data scaling can be distinguished, particularly dispersion-based (based on standard deviation) and central tendency-based measures (based on e.g., mean) (van den Berg et al., 2006).

Autoscaling and Pareto scaling which use dispersion measure, constitute the most commonly applied methods in metabolomics studies. The autoscaling, also known as unit or unit variance (UV) scaling uses the standard deviation as the scaling factor. As a result of UV scaling, all variables have a standard deviation equal to one, so that the transformed dataset is analyzed based on correlations instead of covariances (van den Berg et al., 2006). The Pareto scaling, for which the square root of the standard deviation is applied as the scaling factor, is very similar to the UV scaling. As a result of the Pareto scaling, large fold changes are decreased more than small fold changes and therefore the large fold changes are less dominant as compared to the raw data (van den Berg et al., 2006).

#### Least absolute shrinkage and selection operator (LASSO)

The concept of regularization (also known as penalization) was initially proposed by Tikhonov to solve integral equations (1943) (Kalivas, 2012). The LASSO algorithm was introduced into the field of statistics by Tibshirani (1996). Apart from LASSO, regularization-based methods cover ridge regression, elastic net, bridge regression, and their extensions as well (Ogutu and Piepho, 2014). In its original form, the LASSO method estimates the value of $\beta_{j}^{\prime }$ regression coefficients by minimizing the following objective function (1):

(1) $$\begin{array}{l} LASSO={\left(\overset{n}{\underset{i\;=\;1}{\sum }}y_{i}^{\prime }-\beta_{0}^{\prime }-\overset{p}{\underset{j\;=\;1}{\sum }}\beta_{j}^{\prime }x_{ij}^{\prime }\right)}^{2}+\lambda \overset{p}{\underset{j\;=\;1}{\sum }}|\beta_{j}^{\prime }| \end{array}$$

(2) $$\begin{array}{l} \overset{p}{\underset{j=1}{\sum }}|\beta_{j}^{\prime }|\leq \lambda \end{array}$$

where $\beta_{j}^{\prime }$ represents a standardized regression coefficients, β_0_′ is the intercept, *y*_*i*_′ is the continuous response variable for *i* individual, $x_{ij}^{\prime }$ is the matrix of standardized covariates, *p* denotes a predictor variable, *n* refers to sample size and λ is a tuning parameter (also known as penalty term).

Considering the binary response variable, the log-likelihood function used in classical logistic regression (3) is reconstructed after applying the penalty term (2) to form LASSO penalized logistic regression (Pineda et al., 2014) (4).

(3) $$\begin{array}{ll} & \ln\;L\left(y_{i},\beta \right)=\overset{n}{\underset{i\;=\;1}{\sum }}\left[y_{i}\ln\;\left(\frac{\pi_{i}}{1-\pi_{i}}\right)\right]+\overset{n}{\underset{i\;=\;1}{\sum }}\ln\;\left(1-\pi_{i}\right) \end{array}$$

(4) $$\begin{array}{l} g\left(y_{i};\beta ;\lambda \right)=L\left(y_{i},\beta \right)+\lambda \overset{p}{\underset{j\;=\;1}{\sum }}|\beta_{j}| \end{array}$$

The LASSO assumes sparse solution which means that some regression coefficients are penalized more and some are penalized less toward zero. The tuning parameter λ controls the amount of shrinkage imposed on regression coefficients according to (2). If λ is large, coefficients are penalized highly toward zero (all absolute coefficients are penalized). Low value of λ imposes little penalty on the coefficients (least square criterion is assumed). The most common technique to estimate λ is cross-validation, however other criteria also exist (e.g., AIC, *Akaike Information Criterion*; BIC, *Bayesian Information Criterion*). Considering large sample space, the advantage of LASSO lies in the development of more stable models via reduction of variance, however at the cost of biased estimates.

We can distinguish between three regularization methods: the least absolute shrinkage and selection operator (LASSO) and ridge regression, which are based on the one-norm (L1) and two-norm (L2) minimizations, respectively. The third method constitutes a combination of ridge and LASSO and is known as the naive elastic net.

The L1 and L2 regularization assumes shrinkage of coefficients toward zero to prevent model overfitting introduced either by collinearity of variables or high-dimensionality. The amount of shrinkage assumed by L1 is greater resulting in many regression coefficients shrunken toward zero. In contrast, L2 penalization leads to small but non-zero regression coefficients. Combining L1 and L2 penalties (naive elastic net) tends to give a result in between (Goeman et al., 2014). We used “penalized” package in R (R Core Team, 2014) to fit the LASSO model.

## Results

**Figure 1:**
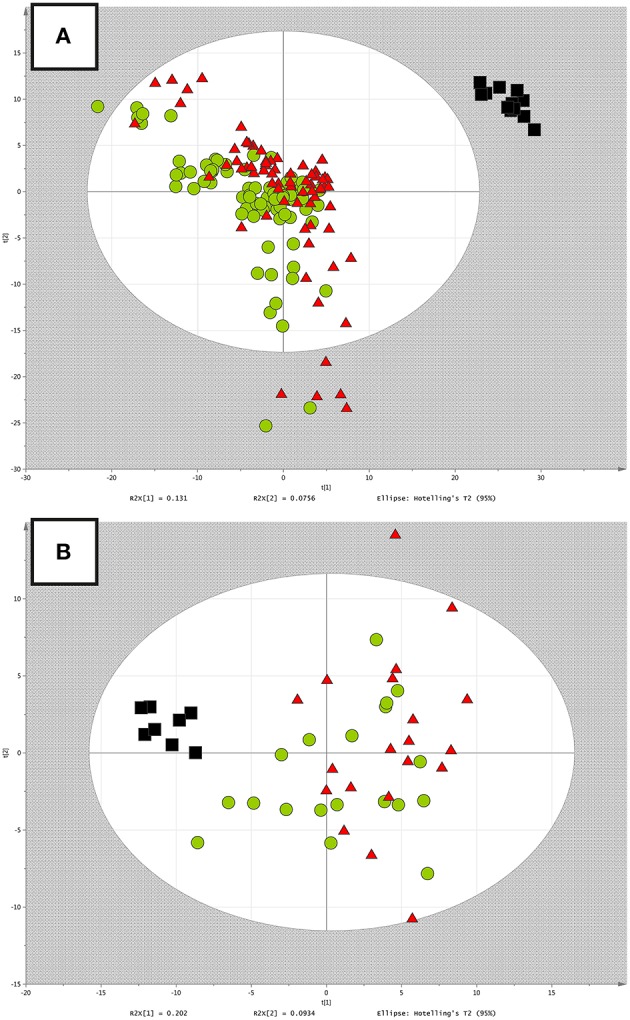
**(A)** PCA model built after data filtration in the *RH study*. Red triangles, green circles and black boxes correspond to the non-RH group, RH group, and QC samples, respectively. **(B)** PCA model built after data filtration in the *PH study*. Red triangles, green circles and black boxes correspond to the PH group, control group and QC samples, respectively.

In the first step of the untargeted metabolomics data analysis for both datasets, we used PCA to check the quality of analysis (grouping of QCs), unveil general trends in the data and find potential outliers based on Hotelling's T2 range. In PCA models, clustering of the QCs was observed which confirmed stability of the analytical system and repeatability of the applied method. Additionally, in the *RH study*, 5 samples were found to be strong outliers and therefore were excluded from further statistical analyses. The obtained PCA models were presented in Figure 1.

The OPLS-DA, as a supervised multivariate method, was used to select variables representing the greatest contribution into groups' classification. The quality of each multivariate model developed was described by R^2^ and Q^2^ which corresponded to the model's goodness-of-fit and predictive performance, respectively. The R^2^ and Q^2^ values were calculated based on 7-fold cross-validation. The value of VIP > 1 denoted variables which contributed the most into groups' classification. Both Pareto and UV scaling methods were tested in OPLS-DA model development. Variables which contribute the most into group recognition were identified (http://ceumass.eps.uspceu.es/mediator) and Human Metabolome Database (www.hmdb.ca). The criteria of database searching included: mass error limited to 10 ppm and possible adducts such as: neutral monoisotopic mass, M+H^+^ and M+Na^+^.

### *RH* and *PH study* in terms of OPLS-DA without multiple testing procedure

All the variables after data filtration, were considered when developing the OPLS-DA model. In the *RH study*, as a result of data extraction and alignment, the obtained dataset contained 126.641 measured variables. After filtration based on the QA criteria (which included the presence of the variables in at least 50% of QCs and the coefficient of variation (CV) value <20% in QCs), the dataset was reduced to 1344 and consequently to 650 variables for which the presence in 80% of the samples in at least one of the compared groups, was reported.

In the *PH study*, as a result of the data extraction and alignment, the obtained dataset contained 225.841 measured variables. After filtration based on the QA criteria (which included the presence of the variables in at least 50% of QCs and the coefficient of variation (CV) value <20% in QCs), the dataset was reduced to 1950 and consequently to 838 variables for which the presence in 80% of the samples in at least one of the compared groups was reported.

For the *RH study*, the OPLS-DA model selected 46 and 218 variables based on the VIP criteria using Pareto and UV scaling, respectively. The R^2^ and Q^2^ were equal to 0.92, 0.88, and 0.83, 0.61 for Pareto and UV scaling, respectively.

In the case of the *PH study*, 217 and 320 variables were selected based on the VIP criteria using Pareto and UV scaling, respectively. The R^2^ and Q^2^ were equal to 0.98, 0.53, and 0.92, 0.44 for Pareto and UV scaling, respectively.

**Figure 2:**
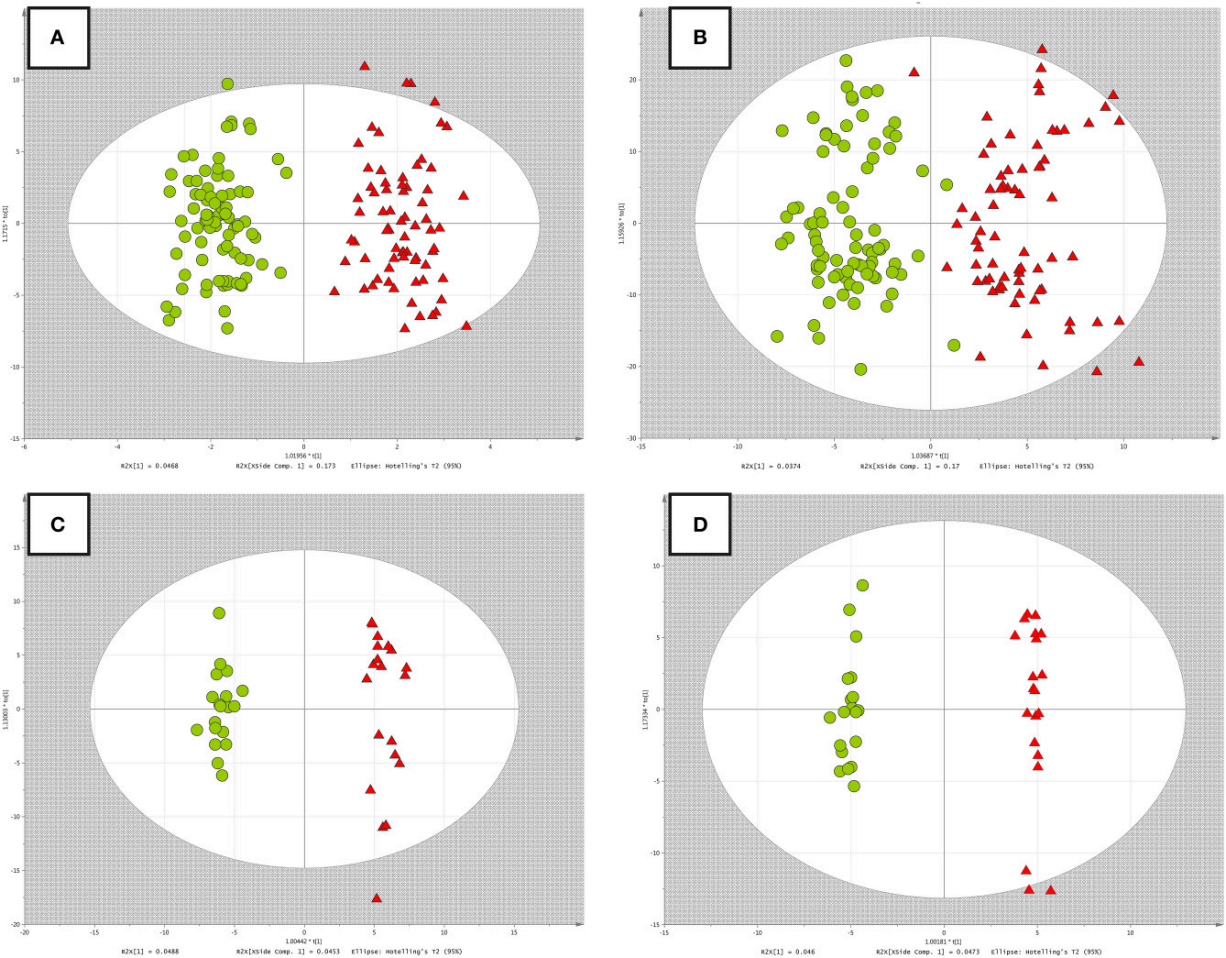
**OPLS-DA models built on data without *multiple testing procedure*. (A)** *RH study*, Pareto scaling; **(B)** *RH study*, UV scaling; **(C)** *PH study*, Pareto scaling; **(D)** *PH study*, UV scaling. Red triangles, green circles correspond to the RH or PH group and non-RH or control group, respectively.

The OPLS-DA models built for both datasets, using different scaling methods, were displayed in Figure 2.

### *RH* and *PH study* in terms of OPLS-DA with multiple testing procedure

In this strategy, we applied FDR correction to pre-select variables and to account for multiple testing. In the *RH* and *PH study*, 62 and 47 variables were statistically significant between investigated group after FDR correction and were further used to develop OPLS-DA models.

Taking into account different scaling procedures and based on VIP criteria, in the *RH study*, 4 and 19 variables were selected as statistically significant in terms of Pareto and UV scaling, respectively. The R^2^ and Q^2^ of OPLS-DA models were equal to 0.47, 0.39, and 0.46, 0.41 for Pareto and UV scaling, respectively.

For *PH study*, 14 and 18 variables were selected as statistically significant based on VIP criteria in terms of Pareto and UV scaling, respectively. The R^2^ and Q^2^ were equal to 0.68, 0.58, and 0.64, 0.52 for Pareto and UV scaling, respectively.

**Figure 3:**
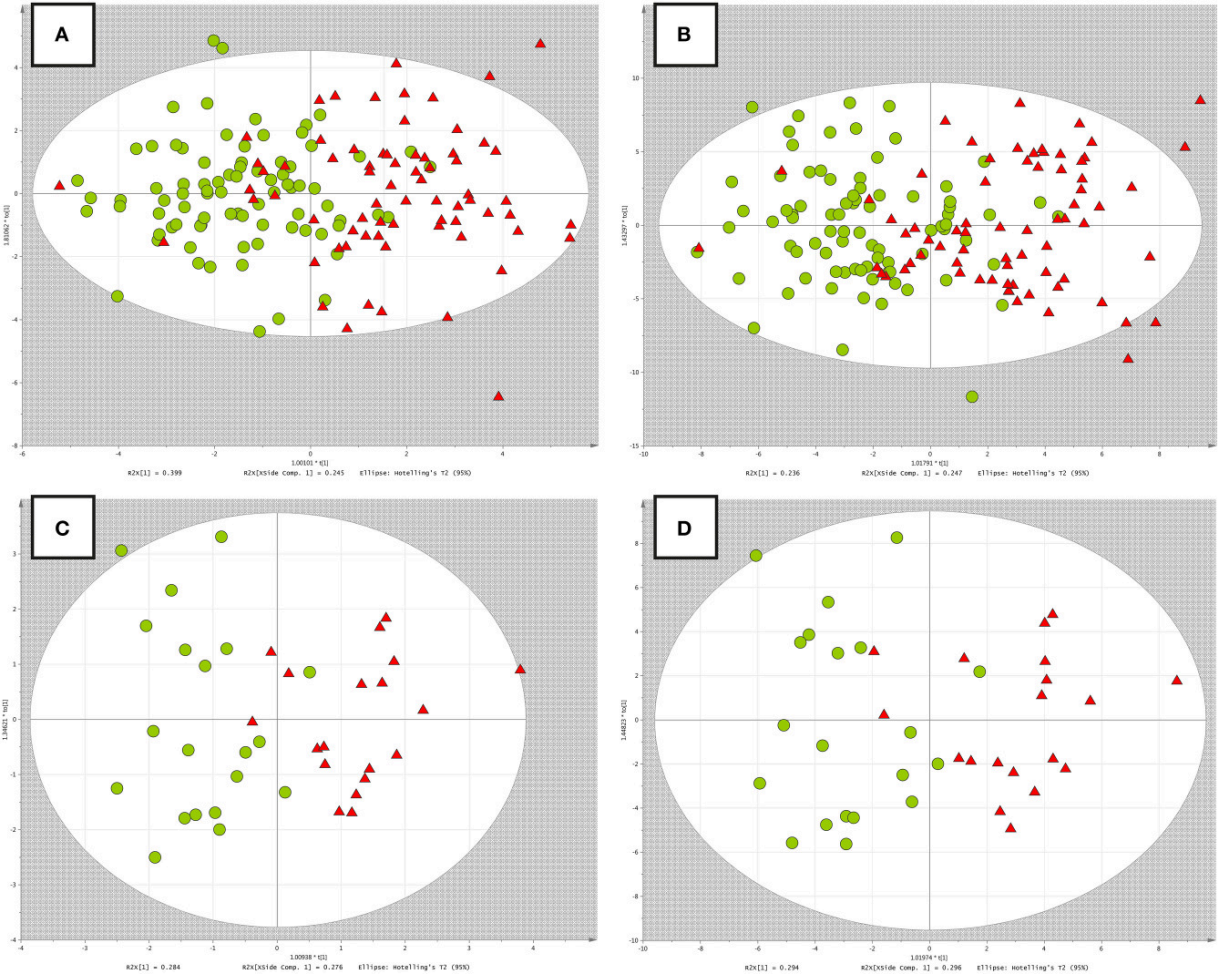
**OPLS-DA models built on data with *multiple testing procedure*. (A)** *RH study*, Pareto scaling; **(B)** *RH study*, UV scaling; **(C)** *PH study*, Pareto scaling; **(D)** *PH study*, UV scaling. Red triangles, green circles correspond to the RH or PH group and non-RH or control group respectively.

The OPLS-DA models built for both datasets, using different scaling methods, were displayed in Figure 3.

### *RH* and *PH study* in terms of LASSO method

For both datasets, we developed the LASSO model with simultaneous 5-fold cross-validation (CV) to select the optimal value of λ for which minimum AIC was obtained. Using this methods, we selected a subset of metabolites, which contributed the most into classification between groups. The variables' coefficients were biased, thus no statistical significance (*p*-value) can be provided, as the standard errors cannot be calculated under a biased estimator. For this reason, the robustness of each metabolite selected in the LASSO model was evaluated using the resampling-based bootstrap procedure. This procedure assumed generation of 1000 resamples for which, the LASSO model was developed. The reproducibility of the results was calculated as a proportion (per 1000 times) each metabolite was introduced into the model.

In the *RH study*, out of 650 variables, the LASSO algorithm selected 14 variables with non-zero coefficients with the corresponding reproducibility for each metabolite ranging from 99.3 to 100%. Among 14 metabolites selected, 11 were found in metabolomics databases. Out of 11 metabolites mentioned earlier, 6 represented known biochemical role.

In the *PH study*, out of 838 variables, the LASSO algorithm selected 4 variables with non-zero coefficients with the corresponding reproducibility for each metabolite ranging from 91.4 to 94.6%. Among 4 metabolites selected, 2 were found in metabolomics databases. Out of 2 metabolites mentioned earlier, only 1 represented known biochemical role.

**Figure 4:**
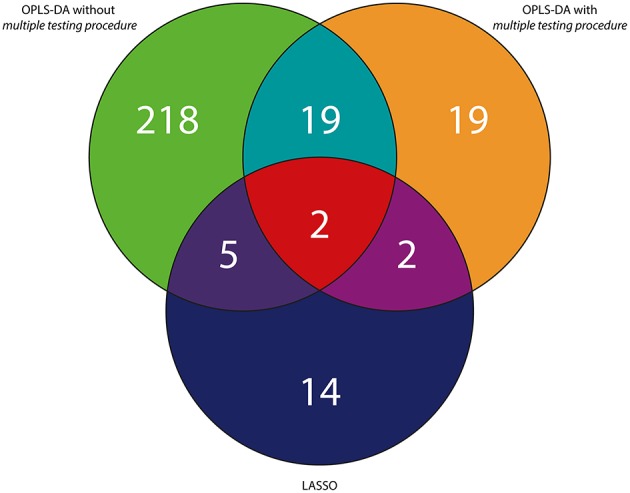
**Venn diagram representing the summary of variables selected with the use of three different approaches exemplified by the UV scaling in case of OPLS-DA for the *RH study***.

**Figure 5:**
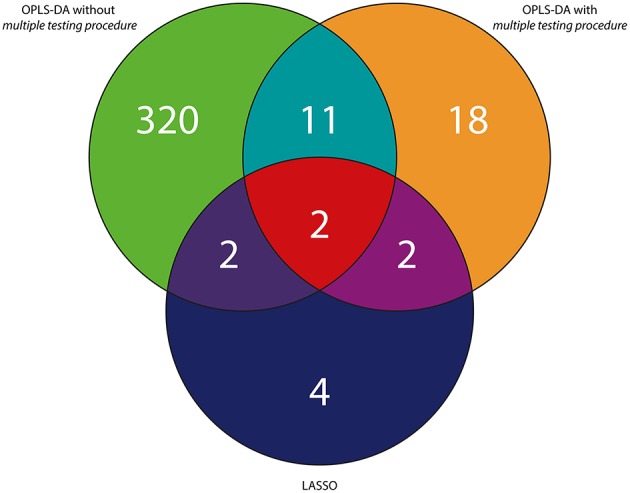
**Venn diagram representing the summary of variables selected with the use of three different approaches exemplified by the UV scaling in case of OPLS-DA for the *PH study***.

To sum up, considering the *RH and PH study*, 2 variables were statistically significant and were found to be in common in three tested approaches (Figures 4, 5). In case of the *RH study*, selected features were identified in publicly available databases as decanamide and C16 sphinganine. In case of the *PH study*, selected features were identified in publicly available databases as tryptophan and palmitoylcarnitine. The results of statistical analyses using three different approaches as well as putative identification of the selected variables were collected in Tables 1, 2, as well as in Tables S3, S4 in the Supplementary Material.

**Table 1:** **Results of statistical analyses using three different approaches and putative identification of the selected variables in case of the *RH study***.

**Variables**	**OPLS-DA without** ***a priori*** **variable selection**		**OPLS-DA with** ***a priori*** **variable selection**		**LASSO**
	**Pareto scaling**	**UV scaling**	**Pareto scaling**	**UV scaling**	
Selected variables	46	218	4	19	14
Found in databases	35	120	3	10	11
Involved in biochemical pathways	20	54	2	7	6

**Table 2:** **Results of statistical analyses using three different approaches and putative identification of the selected variables in case of the *PH study***.

**Variables**	**OPLS-DA without** ***a priori*** **variable selection**		**OPLS-DA with** ***a priori*** **variable selection**		**LASSO**
	**Pareto scaling**	**UV scaling**	**Pareto scaling**	**UV scaling**	
Selected variables	217	320	14	18	4
Found in databases	112	179	8	10	2
Involved in biochemical pathways	82	89	6	8	1

## Discussion

### Considerations on modeling techniques used in the study

In the present study, we compared the OPLS-DA models built on the LC/MS-based datasets without and with multiple testing correction. Additionally, we presented the concept of the LASSO for the analysis of large (*n* = 150) and quite small (*n* = 40) metabolomics data. Selection of metabolites, which contribute the most into group classification constitutes a crucial step in metabolomics research. However, there is no universal and ideal method dedicated for statistical analysis in non-targeted metabolomics approach.

The proportion between the number of observations (samples) and variables should always be considered. The sample size in non-targeted metabolomics studies is usually small as compared to the number of variables. Therefore, the use of any variable selection method before multivariate model development should be consider to reduce the curse of dimensionality, avoid overfitting of the model *via* reduction of false positive findings and consequently providing generalization of the developed model (Bum Kim et al., 2008). There are few methods which account for multiple testing e.g., FDR, Bonferroni correction, which are often applied in metabolomics-based experiments to avoid false discoveries and to remove irrelevant variables (Broadhurst and Kell, 2006). At this point, it should be noted, that Bonferroni correction is considered the most stringent and assumes independency of the variables tested, which may not be the case for metabolomics studies.

According to the available literature, there are numerous approaches of feature selection based on orthogonal projections: recursive algorithm, support vector machine, genetic algorithm, or random forest, which aim at selecting spectral features contributing the most into class separation (Ramadan et al., 2006; Wongravee et al., 2009; Lin et al., 2011). The MS-based metabolomics datasets usually contain a large number of variables, of which only a small proportion could be considered relevant. The PLS-DA and OPLS-DA as multivariate discriminant methods, are the most commonly applied in non-targeted metabolomics studies (Holmes et al., 2008; Triba et al., 2015).

In the present study, prior the development of OPLS-DA model, both Pareto and UV scaling were tested. For the *RH* and *PH study*, a far more variables were selected when the UV scaling was applied. As a result of the UV scaling, all variables (metabolites) became equally important. The Pareto scaling in turn, is more sensitive to large fold changes and therefore variables with lower fold changes may be treated as irrelevant. It can be the reason why more variables were selected in the OPLS-DA models when the UV scaling was implemented. Data scaling is an important step before PLS-DA and OPLS-DA multivariate statistical analysis, aimed at providing a proper selection of relevant variables. There are many different scaling methods which can be applied (e.g., vast, range, level etc.) and detailed information describing their advantages and disadvantages can be found in the literature (van den Berg et al., 2006; Gromski et al., 2015). Moreover, it should be underlined that data scaling has a great influence on accuracy of the classification model in metabolomics studies. It can be concluded that data scaling before multivariate analysis may affect the selection of relevant variables in non-targeted metabolomics experiments. Therefore, testing toward various scaling methods is recommended to be performed and compared.

However, apart from popularity of PLS-DA and OPLS-DA methods in metabolomics, it should be highlighted that they do not control the type I or type II errors, but only arbitrarily establish a cut-off value for the loadings. Such multivariate model represents high goodness-of-fit to the data, however the risk of overfitting increases relevantly.

Therefore, in this study we proposed and implemented the concept of LASSO to perform variable selection and model development simultaneously. When modeling a binary outcome, the LASSO algorithm uses the log-likelihood function (similar as for the logistic regression) together with the penalty term. The resulting LASSO penalized logistic regression is then capable of shrinking the coefficients toward zero which is not possible when applying the maximum likelihood alone. The shrinkage reduces the variance at the cost of bias of coefficients which is known to improve prediction performance of the model, especially when we deal with the so-called “small *n* large *p*” problem. The LASSO operates by including *n* variables into the model and selecting only those which are mostly associated with the response (Tibshirani, 1996). Therefore, the sparsity assumption offered by the LASSO helps recovering the underlying signal from the high dimensional data.

As mentioned earlier, the strength of LASSO lies in the variable selection, however it should be noted that it is not applicable when grouped selection for strongly correlated predictors is the case. In contrast, ridge regression accounts for the grouped selection. A compromise between the ridge regression and LASSO is served by the elastic net. It builds a regression model penalized with both the L1-norm and L2-norm, which results in shrinking coefficients (as in ridge regression) and penalizing some of them toward zero (as in LASSO). In contrast to ridge regression, elastic net provides sparse estimates of the coefficients (Tibshirani, 1996).

In this study, instead of calculating the standard errors for biased coefficients, we used bootstrap to assess the reproducibility of the results. We obtained high robustness for selected metabolites and therefore we can conclude, that this method may be considered for untargeted metabolomics study. The number of variables selected via LASSO in the *RH* and *PH study* is much lower in comparison to the OPLS-based methods and this results from the basic principles of this method and the presence of the constrain.

### Biological considerations on discriminating features

In the *RH study*, the discriminating features selected by three different approaches, were putatively identified as decanamide and C16 sphinganine. Decanamide constitutes an example of free fatty acid amides (FFAMs) which can be a product of two different routes, i.e., ammonolysis of fatty acyl-CoA thioesters and oxidative cleavage of *N* -fatty acylglycines (Farrell et al., 2012). Recent studies on FFAMs have indicated their importance as signaling molecules involved in various biological processes such as sleep, motion, angiogenesis, release of Ca^2+^ and blood vessels relaxation (Farrell et al., 2012). The C16 sphinganine is a ceramide-related sphingolipid. Sphingolipids are a major class of lipids employed in eukaryote membranes composition, especially in the central system, however they constitute bioactive signaling molecules playing a crucial role in cell growth, apoptosis, signal transduction, and recognition (Bartke and Hannun, 2009).

In case of the *PH study*, the discriminating features selected by three different approaches, were putatively identified as tryptophan and palmitoylcarnitine. Tryptophan is amino acid converted into serotonin by tryptophan hydroxylase enzyme. Serotonin has been suggested to enhance pulmonary arterial smooth muscle cell proliferation, vasoconstriction and microthrombosis (MacLean et al., 2000). The second identified metabolite, namely palmitoylcarnitine belongs to the group of long-chain acylcarnitines, which facilitates the transfer of long-chain fatty acids from the cytoplasm to mitochondria during fatty acid β-oxidation (FAO). The changes of palmitoylcarnitine in plasma level reported in this study, may suggest incomplete FAO, which might be associated with impairment of the tricarboxylic acid cycle known to occur in pulmonary diseases (Archer et al., 2013).

## Conclusions

High-dimensional data space is a domain of untargeted metabolomics. The larger the search space, the higher the probability of developing models that are well fitted to the training data, even though they might not have any predictive performance. This phenomenon may explain the discrepancy between R^2^ and Q^2^ values frequently observed in PLS-based models, leading to high variance of the coefficients and model overfitting. Regularization plays a key role in high-dimensional problems as it assumes sparse solutions by imposing a constrain on the coefficients' value, shrinking them toward zero and simultaneously reducing their variance. Such an approach reduces overfitting and thus provides more accurate models offering an alternative to widely used PLS-based methods.

## Author contributions

RB has conducted PLS-based data analysis of metabolomics datasets. EDW has performed LASSO analysis of metabolomics datasets. RB and EDW have written the manuscript. MM has designed the study, reviewed the obtained results and the manuscript. RK has reviewed and corrected the prepared manuscript.

### Conflict of interest statement

The authors declare that the research was conducted in the absence of any commercial or financial relationships that could be construed as a potential conflict of interest.
