# Supplementary material for: PLS-Based and Regularization-Based Methods for the Selection of Relevant Variables in Non-targeted Metabolomics Data
Source: Front Mol Biosci. 2016 Jul 26;3:35. doi: 10.3389/fmolb.2016.00035 (PMC4960252; doi:10.3389/fmolb.2016.00035)
Supplement: Supplementary file 1 [file DataSheet1.doc]

Supplementary Material

**PLS-based and regularization-based methods for the selection of relevant variables in non-targeted metabolomics data.**

**Renata Bujak^1#^, Emilia Daghir-Wojtkowiak^1#^, Roman Kaliszan^1^, Michał Jan Markuszewski^1*^**

**^1^ Department of Biopharmaceutics and Pharmacodynamics, Medical University of Gdańsk, Al. Gen. J. Hallera 107, 80-416, Gdańsk, Poland**

**# Both authors contributed equally**

*** Correspondence:** Michał Jan Markuszewski, markusz@gumed.edu.pl

# Supplementary Data

2.1 Study design and analytical measurements

In the first non-targeted metabolomics study, the HPLC analysis was performed at the temperature of 25°C using Ascentis Express C18 column (15 cm x 4.6 mm, 2.7 µm, Supelco Analytical, USA). Plasma samples (2 μl) were analyzed using scan mode in the range of 100 to 1100 m/z (mass to charge ratio) in positive ionization mode. As a mobile phase, 0.1 % formic acid (97%, Alfa Aesar, Germany) in deionized water (A) and 0.1 % formic acid in acetonitrile (B), were used. A flow rate of 0.35 ml/min was applied. The following gradient program was carried out from 0 to 25 minutes, the mobile phase B eluted from 2 % to 98 % and then was kept for 10 min at 98 % of B. Additionally, we established an equilibration time at 12 min. The scan rate was set to 1.51 spectra/second and the data acquisition time to 662.3 ms/spectrum. To provide accurate mass measurements, two reference masses (namely 121.0509 and 922.0098 m/z) were automatically delivered using dual ESI source during plasma sample analyses. Capillary voltage was set to 3250 V and the nebulizer gas flow rate and pressure were 10 L/min and 30 psig, respectively. Fragmentor voltage was set to 150 V. All plasma samples were analyzed in a random order.

In the second non-targeted metabolomics study, extracted plasma samples (10 μL) were injected onto a reversed-phase column (Discovery HS C18, 15 cm x 2.1 mm, 3 µm; Supelco) with a pre-column (Discovery HS C18; 2cm x 2.1 mm, 3 mm; Supelco) thermostated at 40 °C. The system was operated in the positive and negative modes at 0.6 mL/min flow rate with solvent A, water with 0.1% formic acid, and solvent B, acetonitrile with 0.1% formic acid. The gradient started from 25% B to 95% B in 35 min, and returned to initial conditions in 1 min, keeping the re-equilibration at 25% B for 9 min. The detector operated in a full scan mode from 50 to 1000 m/z with a scan rate of 1 scan per second. Accurate mass measurements were obtained by means of an automated calibrant delivery system using dual ESI source which continuously introduced a calibration solution, with reference masses at m/z 121.0509 (protonated purine) and m/z 922.0098 [protonated hexakis(1H,1H,3Htetrafluoropropoxy) phosphazine or HP-921]. The capillary voltage was set to 3000 V and the nebulizer gas flow rate was 10.5 L/min. Samples in a random order were analyzed in a sequence run in the positive ionization mode.

Table S1. The detailed clinical data of compared groups in the *RH study*.

|  | Non-resistant hypertension (Non-RH)  n=81 (W=27, M=54) | | | | Resistant hypertension (RH) n=69 (W=23, M=46) | | | | |
| --- | --- | --- | --- | --- | --- | --- | --- | --- | --- |
|  | Mean | Min | Max | SD | Mean | Min | Max | SD |  |
| Age [years] | 57.74 | 19.00 | 83.00 | 12.77 | 58.25 | 38.00 | 81.00 | 11.29 |
| SBP [mmHg] | 127.52 | 102.74 | 177.95 | 10.61 | 149.86 | 137.89 | 189.91 | 11.63 |
| DBP [mmHg] | 74.23 | 54.57 | 110.08 | 9.13 | 86.48 | 65.45 | 119.48 | 9.63 |
| BMI [kg/m^2^] | 30.40 | 19.90 | 42.60 | 4.75 | 31.27 | 21.80 | 44.00 | 5.15 |
| Waist [cm] | 103.52 | 74.00 | 134.00 | 13.05 | 105.88 | 78.00 | 132.00 | 13.35 |
| Hip [cm] | 107.62 | 87.00 | 132.00 | 8.94 | 109.39 | 92.00 | 134.00 | 8.88 |
| WHR | 0.96 | 0.76 | 1.15 | 0.08 | 0.97 | 0.80 | 1.14 | 0.08 |
| eGFR [ml/(min×1.72m^2^] | 90.20 | 43.65 | 147.12 | 18.13 | 82.75 | 37.29 | 122.30 | 17.87 |
| Number of drugs (n) | 3.94 | 2.00 | 6.00 | 1.12 | 4.41 | 3.00 | 7.00 | 1.17 |
| Smoking (n) | 12 | | | | 8 | | | |
| Dyslipideamia (n) | 66 | | | | 57 | | | |
| CVD (n) | 6 | | | | 26 | | | |
| DM (n) | 28 | | | | 18 | | | |

eGFR - estimated glomerular filtration rate, SBP/DBP - systolic/diastolic blood pressure obtained from 24-hour ambulatory blood pressure monitoring, BMI - body mass index, Waist/Hip – circumference, WHR - waist to hip ratio, PWV - pulse wave velocity, CVD - cardiovascular disease, DM - diabetes mellitus type 1 or 2, Smoking - status of current tobacco smoking, N – number, Min - minimum value, Max - maximum value, SD - standard deviation, W – women, M – man.

Table S2. The detailed clinical data of compared groups in the *PH study*.

| **Patient**  **ID** | **Age** | **Gender** | **BMI** | **PAH**  **etiology** | **Hepatitis** | **HIV**  **infection** | **DM** | **Nephritis** | **IHC** | **AVC** |
| --- | --- | --- | --- | --- | --- | --- | --- | --- | --- | --- |
| PH 1 | 22 | F | 19.7 | 1 | 0 | 0 | 0 | 0 | 0 | 0 |
| PH 2 | 38 | F | 21.9 | 1 | 0 | 0 | 0 | 0 | 0 | 0 |
| PH 3 | 66 | F | 25.4 | 2 | 0 | 0 | 0 | 1 | 0 | 0 |
| PH 4 | 67 | F | 31.4 | 2 | 0 | 0 | 0 | 0 | 0 | 0 |
| PH 5 | 60 | M | 27.1 | 2 | 0 | 0 | 0 | 0 | 1 | 0 |
| PH 6 | 30 | F | 25.0 | 1 | 0 | 0 | 0 | 0 | 0 | 0 |
| PH 7 | 59 | F | 27.0 | 2 | 0 | 0 | 0 | 0 | 0 | 0 |
| PH 8  PH 9  PH 10  PH 11  PH 12  PH 13  PH 14  PH 15  PH 16  PH 17  PH 18  PH 19  PH 20  CONTROL 1  CONTROL 2  CONTROL 3  CONTROL 4  CONTROL 5  CONTROL 6  CONTROL 7  CONTROL 8  CONTROL 9  CONTROL 10  CONTROL 11  CONTROL 12  CONTROL 13  CONTROL 14  CONTROL 15  CONTROL 16  CONTROL 17  CONTROL 18  CONTROL 19  CONTROL 20 | 57  24  62  70  47  45  58  57  44  31  51  73  54  23  36  63  67  60  31  57  57  23  58  69  49  45  60  59  44  31  51  72  51 | M  F  F  F  F  F  F  M  M  F  F  M  F  F  F  F  F  M  F  F  M  F  M  F  F  F  F  M  F  F  F  M  F | 29.0  27.0  31.6  25.5  22.6  23.8  20.7  30.1  22.2  19.6  25.0  29.7  22.9  21.4  20.0  26.0  31.2  27.3  22.7  28.2  28.6  25.0  31.8  24.5  23.7  23.9  22.4  28.4  20.8  19.6  24.1  30.0  23.7 | 1  3  2  2  5  2  4  6  1  1  1  1  1  0  0  0  0  0  0  0  0  0  0  0  0  0  0  0  0  0  0  0  0 | 0  0  0  0  1  0  0  0  0  0  0  0  0  0  0  0  0  0  0  0  0  0  0  0  0  0  0  0  0  0  0  0  0 | 0  0  0  0  1  0  0  0  0  0  0  0  0  0  0  0  0  0  0  0  0  0  0  0  0  0  0  0  0  0  0  0  0 | 0  0  0  0  0  0  0  1  0  0  0  0  0  0  0  0  1  0  0  0  0  0  0  0  0  0  1  0  0  0  0  0  0 | 0  0  0  0  0  0  0  0  0  0  0  0  1  0  0  0  0  0  0  0  0  0  0  0  0  0  0  0  0  0  0  0  0 | 0  0  0  0  0  0  0  0  0  0  0  0  1  0  0  0  0  0  0  0  0  0  0  0  0  0  0  0  0  0  0  0  0 | 0  0  0  0  0  0  0  0  0  0  0  0  0  0  0  0  0  0  0  0  0  0  0  0  0  0  0  0  0  0  0  0  0 |

In case of gender: M denotes man, F denotes woman.

In case of the PAH etiology: 0-control, 1-idiopathic, 2-PAH due to systemic sclerosis, 3-PAH due to other connective tissue diseases, 4-PAH due to congenital heart diseases, 5-PAH due to HIV infection, 6- portopulmonary hypertension (POPH), 7-PAH due to schistosomiasis.

In other cases: 1 means presence and 0 means absence of the disease, DM-diabetes mellitus, IHC- ischemic heart disease, AVC- atrioventricular canal defect.

Table S3. Identification of metabolites selected by three different approaches in the *RH study*.

| Measured mass | Mass in database | Mass error | Retention time | Name | Formula |
| --- | --- | --- | --- | --- | --- |
| 171.1624 | 171.1623 | -0.6 | 22.0 | decanamide | C10H21NO |
| 273.2661 | 273.2668 | 2.6 | 19.1 | C16 sphinganine | C16H35NO2 |

Table S4. The identification of metabolites selected by three different approaches in the *PH study*.

| Measured mass | Mass in database | Mass error | Retention time | Name | Formula |
| --- | --- | --- | --- | --- | --- |
| 204.0897 | 204.0899 | 0.9 | 1.1 | Tryptophan | C11H12N2O2 |
| 399.3347 | 399.3349 | 0.5 | 18.1 | Palmitoylcarnitine | C23H45NO4 |
